# Prevalence of *aac(6′)-Ie-aph(2″)-Ia* resistance gene and its linkage to Tn5281 in *Enterococcus faecalis* and *Enterococcus faecium* isolates from Tabriz hospitals

**Published:** 2013-09

**Authors:** Amir Behnood, Safar Farajnia, Seyed Reza Moaddab, Shiva Ahdi-Khosroshahi, Aliakbar Katayounzadeh

**Affiliations:** 1 Biotechnology Research Center; 2 Drug Applied Research Center; 3 Paramedical Faculty; 4 Tuberculosis and Lung Disease Research Center; 5 Research Center for Infectious and Tropical Disease, Tabriz University of Medical Sciences, Tabriz, Iran

**Keywords:** Enterococci, High-level gentamicin resistance (HLGR), Long PCR, Dot-Blot hybridization

## Abstract

**Background and Objective:**

High-level gentamicin resistance (HLGR: MIC ≥ 500 µg/ml) in Enterococci is mediated by aminoglycoside modifying enzymes which is mainly encoded by *aac(6′)-Ie-aph(2″)-Ia* gene. The aim of this study was to evaluate the frequency of *aac(6′)-Ie-aph(2″)-Ia* gene in clinical isolates of *Enterococcus facium* and *Enterococcus faecalis* collected from hospitals in northwest of Iran.

**Materials and methods:**

In the present study a total of 111 enterococcus isolates were collected from 4 hospitals during a two year period (July 2009-August 2011). Bacterial identification and species determination were carried out by standard biochemical tests. Antimicrobial susceptibility was evaluated by Kirby Bauer disc diffusion method. MICs were determined by agar dilution method. The frequency of *aac(6′)-Ie-aph(2″)-Ia* gene in the isolates was determined by PCR. The carriage of resistance gene on Tn5281 transposon was identified by long PCR and dot-blot hybridization methods.

**Results:**

Antibiotic susceptibility tests revealed that the highest resistance was against streptomycin (74.77%) and erythromycin (67.58%) whereas the highest susceptibility was observed to vancomycin (81.1%). 36 isolates (32.43%) were identified as HLGR, 34(94.44%) of them had resistant gene in their genome. Long PCR studies revealed that 88% of HLGR clinical isolates harboured Tn5281. The *aac(6′)-Ie-aph(2″)-Ia* resistance gene was present on Tn5281 transposon in all 32 isolates according to dot blot hybridization test.

**Conclusion:**

The results of this study indicated that *aac(6′)-Ie-aph(2″)-Ia* resistance gene is highly prevalent in gentamicin resistant isolates. Carriage of *aac(6′)-Ie-aph(2″)-Ia* resistance gene on Tn5281 transposable element suggests possible contribution of this transposone on dissemination of resistance gene among enterococcus isolates.

## INTRODUCTION

*Enterococcus* (*E*) *faecalis* and *E. faecium* are normal floras of human and animals digestive system, however they are also known as occasional human pathogens responsible for community-acquired and nosocomial infections (1). These bacteria are found in the gastrointestinal and female urinary tracts as part of the normal host flora in healthy individuals where they cause infections (1, 2). Current studies show that these organisms have emerged as a leading cause of nosocomial infections in hospitals. Most of the enterococcal infections are due to *E*. *faecalis* isolate. *E. faecium* is responsible for minority of enterococcal infections (3). Clinical treatments for serious enterococcal infections require a combination of a cell wall active agent and an aminoglycoside, typically gentamicin (4, 5). Enterococcus species can acquire high-level resistance to a variety of antibiotics by horizontal transfer of mobile genetic determinants, in addition to the intrinsic resistance to several groups of antimicrobials (6).

Resistance to the aminoglycosides usually occurs by enzymatic modification of the antibiotics by aminoglycoside-modifying enzymes (AME) (7). The first case of high-level gentamicin-resistant (HLGR) *E. faecalis* isolate was reported by Thal and his colleagues (1979) in France (8). Further investigations showed that the reason for high-level resistance to gentamicin antibiotics was due to the fusion of two aminoglycoside-modifying enzyme genes. The resultant bifunctional *aac(6')-aph(2″)* aminoglycoside modifying enzyme confers resistance to all clinically useful aminoglycosides except streptomycin. The genes responsible for high-level aminoglycoside resistance have in most cases been identified on plasmids (9). Previous investigations have reported the *aac(6')-aph(2″)* gene as being part of a transposable element Tn*5281* (10, 11). Relation of *aac(6')-aph(2″)* with a transposon facilitates rapid distribution of the resistance gene. The aim of this study was to investigate the prevalence of HLGR in clinical isolates of *E. faecalis* and *E. faecium* and to determine the molecular basis of the gene responsible for HLGR in these isolates.

## MATERIALS AND METHODS

### Bacterial isolates

A total of 111 *Enterococcal* isolates were collected from 4 different hospitals in Tabriz, northwest of Iran during July 2009 to August 2011. The identification of the isolates was confirmed with standard biochemical tests. *E. faecalis* strain HH22, containing the HLGR-conferring transposon Tn*5281*, and *E. faecalis* ATTCC 29212 were used as a positive and negative controls, respectively (12).

### Susceptibility testing

Antibiotic susceptibility was determined by Disc diffusion method (Kirby-Baur) according to the CLSI guidelines (13). The following antibiotics were tested: streptomycin, erythromycin, ciprofloxacin, nitrofurantoin, ampicillin, tetracycline, penicillin, vancomycin, and gentamicin. 120 μg gentamicin discs (MAST, UK) were used for identification of high level gentamicin resistant strains. The MIC was determined by agar dilution method. High-level gentamicin resistance was defined as MIC ≥ 500 µg/ml. Multidrug resistance (MDR) was defined as resistance to three or more antimicrobial classes.

### Detection of *aac6*′*-aph2*″ by PCR

PCR was performed to amplify the bifunctional *aac(6')-aph(2″)* gene sequences. Bacterial cells were lysed and DNA extracted as described by Regnault (14). Primers 5'CCAAGAGCAATAAGGGCATA3'and 5'CACTATCATAACCACTACCG3' were used as forward and reverse primers to obtain PCR product of approximately 400 bp. PCR master mix components were as follows; 10X PCR buffer in final concentration of 1X, MgCl_2_ (50mM) in a final concentration of 1.5 mM, dNTP Mix, 10 mM in a final concentration of 0.2 mM and forward and reverse primers in a final concentration of 0.4 μM. PCR amplification was performed in a total volume of 25 μl. Thermal cycling was performed by initial denaturation at 94°C for 4 min followed by 35 cycles of 60 seconds denaturation at 94°C; 60 seconds annealing at 65°C; 45 seconds extension at 72°C with a final extension at 72°C for 7 minutes. PCR products were analyzed by electrophoresis in 1.2% agarose gel and visualized by gel documentation system (Uvitec, UK) after staining with ethidium bromide (15).

### Plasmid extraction

For isolation of plasmid harboring Tn528 transposone, all isolates that were positive for *aac(6')-aph(2″)* gene subjected to plasmid extraction following the method described by Woodford et al (16).

### Long PCR

To identify the isolates containing Tn5281 transposone, a long PCR was carried out with a single primer 5’-CAGAACAGCTGGATCCTATGG-3’ using plasmid extracts as templates. The reaction mixture for long PCR was prepared according to the instructions of manufacturer (Roche diagnostics, Germany). The thermal cycling for amplification of Tn5281 transposone has shown in Table 1. Products were analyzed by gel electrophoresis and photographed to determine the 3.4Kb product.

**Table 1:** Thermal cycling condition for amplification of Tn5281 transposone by long PCR.

Stage	Temperature	Time	Cycles
Initial denaturation	94°C	4 min	1
Denaturation	94°C	10 s	
Annealing	57°C	30 s	
Extension	68°C	45 s	10
Denaturation	94°C	15 s	
Annealing	57°C	30 s	25
extension	68°C	45 s	
Final extension	68°C	7 min	1
Cooling	4°C	10 min	

### Dot-Blot hybridization

Dot-Blot hybridization method was performed to determine the presence of *aac(6')-aph(2″)* gene on Tn5281 transposone. 1 μg of plasmid extracts was dissolved in double distilled water to a final volume of 16 μl and denaturized by heating in a boiling water bath for 10 minutes. 4 μl of digoxigenin labeled *aac(6')-aph(2″)* PCR product was mixed with denaturized plasmid and the mixture was incubated for 1 h at 37°C. Finally the reaction was stopped by adding 2 μl of 200mM EDTA. A serial dilution of hybridization reactions were applied to a positively charged nylon membrane. Part of the nylon membrane was preloaded with defined dilutions of labeled control DNA which were used as standard. The nylon membrane was subjected to immunological detection with anti-digoxigenin antibody. The intensities of hybridization products and control DNA were compared by exposure to imaging device (17).

## RESULTS

### Patients and specimens

**Fig. 1:**
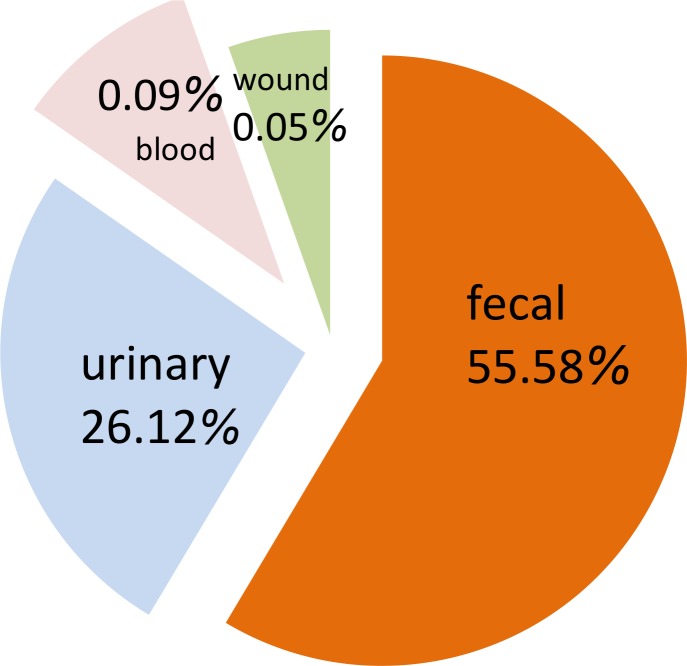
Distribution of clinical specimens used for isolation of entrococci.

In this study, 193 clinical specimens from 90 in-patients and 103 out-patients were collected. A total of 111 Enterococci isolates were isolated from 45 in-patients and 66 outpatients. The origin of isolates has shown in Fig. 1. 89 (80.1%) isolates were identified as *E. faecalis* and 22 (19.8%) were *E. faecium*.

**Fig. 2:**
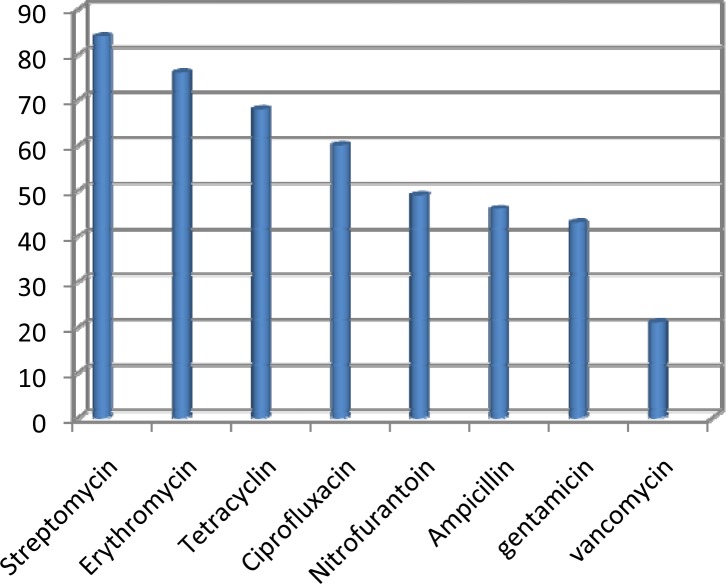
Results of disk diffusion tests for *Enterococcus* isolates. (horizontal line: antibiotics; vertical line: Rate of resistant isolates).

Fig. 2 shows the results of disc diffusion tests. The highest resistance rate was found to streptomycin (74.77%) followed by erythromycin (67.58%), tetracycline (60.36%), ciprofloxacin (53.15%), nitrofurantoin (43.24%), ampicillin (39.64%), gentamicin 43 (38.73%) and vancomycin 21(18.9%).

The results of MIC revealed that 13 (36.11%) *E. faecium* isolates and 23 (63.88%) *E. faecalis* isolates were highly resistant to gentamicin (MIC ≥ 500 µg/ml) (Table 2). Out of 22 *E. faecium* isolates, 6(27.3%) isolates and out of 89 *E. faecalis* isolates, 36 (40.44%) isolates were highly resistant to streptomycin. High level resistances to both streptomycin and gentamicin were found in 5 (36.36%) isolates of *E. faecium* and 14 (15.7%) isolates of *E. faecalis*.

**Table 2:** Results of agar dilution test for detection of high-level gentamicin/ streptomycin resistance in *Enterococcus* isolates.

isolates	n	Gentamicin (MIC ≥ 500 µg/ml) N (%)	Streptomycin (MIC ≥ 2000 µg/ml) N (%)	Streptomycin + Gentamicin (MIC ≥ 2000 µg/ml+ MIC ≥ 500 µg/ml) N (%)
*E. faecalis*	89	23 (25.84)	36 (40.44)	14 (36.36)
*E. faecium*	22	13 (59.09)	6 (27.27)	5 (15.7)

### PCR analysis

**Fig. 3:**
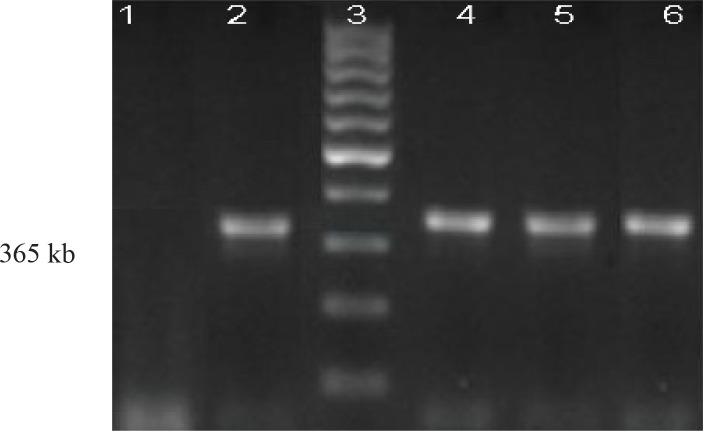
PCR amplification of *aac(6′)-Ie-aph(2″)-Ia* gene from HLGR Enterococci. Line 1: negative control: *E. faecalis ATCC*29212; Line 2: positive control: *E. faecalis HH*22; Lane 3, 1 kb size marker; Line 4–6: isolates with the HLGR genotype.

Among 36 isolates that were identified as HLGR (32.43%), the *aac(6′)-Ie-aph(2″)-Ia* resistance gene was detected in 34(94.4%) isolates. Fig. 3 shows a 365 base pair PCR amplicon associated with high level gentamicin resistance. Two HLGR isolates, were negative for *aac(6')-aph(2″)* gene in PCR method.

### Plasmid analysis and Dot-blot hybridization

**Fig. 4:**
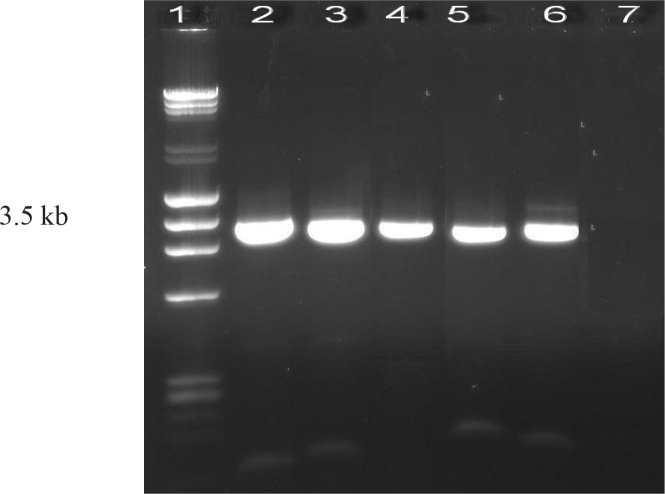
PCR amplification of Tn5281 from HLGR isolates. Lane 1, 1 kb size marker; Lanes 2, 4, 5 and 6: positive clinical isolates; Lane 3: positive control (*E. faecalis* HH22); lane 7: negative control (*E. faecalis* ATCC29212).

**Fig. 5:**
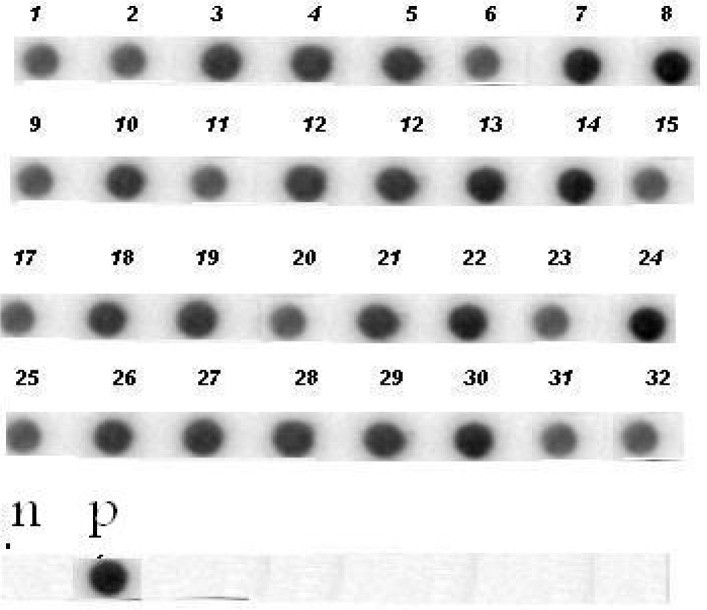
Dot-Blot hybridization of 32 HLGR isolates; P: positive control (*E. faecalis* HH22); N: negative control ( *E.faecalis* ATCC29212); Dots 1 to 32: HLGR isolates.

Results from plasmid analysis showed the presence of a plasmid of about 3.5kb in 94.4% of isolates. Dot-Blot hybridization using *aac(6′)-Ie-aph(2″)-Ia* probe and amplified Tn5281 indicated that *aac(6′)-Ie-aph(2″)-Ia* gene was located on 3.5Kb Transposon in 32(88%) HLGR isolates. Figs. 4 and 5 show the Long PCR amplification of Tn5281 and Dot-blot hybridization results, respectively.

## DISCUSSION

In recent decades enterococci have emerged as a highly important nosocomial and community-acquired pathogens. Although these bacteria are generally thought to be a low virulence pathogen, it is now unveiled that these organisms can cause serious invasive infections, including endocarditis, bacteremia, urinary tract and pelvic infections. The role of enterococci as a causative agent of various infections has become considerably important, not only for their documented pathogenic potential but also due to increasing antimicrobial resistance (especially resistance to glycopeptides) in most of isolates (18). Aminoglycosides are frequently used in combination with cell wall active antibiotics for severe enterococcal infections. It has shown that the cell wall active agents disrupt the bacterial cell wall to allow the aminoglycoside to enter and exert their bactericidal effects (19, 20).

Low-level aminoglycoside resistance is an intrinsic characteristic in all enterococcal species. However, acquired high-level aminoglycoside resistance may be caused by various aminoglycoside-modifying enzymes (19, 20). Several studies demonstrated that aminoglycoside high-level resistance genes in enterococci are encoded on plasmids, conjugative elements, or the most commonly on conjugative transposons, that mediate the horizontal transfer of resistance determinants (9, 18, 19).

In our study, high-level resistance to gentamicin was observed in 32.43% of isolated enterococci. This frequency is lower than the rates reported by Dadfarma (57.4%, 2010) (24) and Feizabadi (65%, 2008) (25). HLGR was reported in 46.15% of isolates in Italy (18), 45.5% in Brazil (26), 82.3% in Michigan (27), 37.64% in Chicago, (28) and in 46.06% in South Africa (29). The lowest rate (15.7%) of HLGR has been reported from Greece (30). These results demonstrate variation of HLGR prevalence in different geographic regions.

High-level resistance to gentamicin and streptomycin was observed in *E. faecium* isolates in 57.1% and 27.3% of cases and in *E. faecalis* in 25.8% and 40.4% of isolates, respectively (Table 2). Multidrug resistance was observed in 49.09% of isolates which is a high rate in comparison to other studies carried out in recent years (18–20).

Resistance to aminoglycosides usually occurs by enzymatic modification of drugs by aminoglycoside modifying enzymes which are carried on mobile elements such as transposons. The results of this study showed that 88% of HLGR isolates contained Tn5281 among them 94% of isolates carried *aac(6′)-Ie-aph(2″)-Ia* resistance gene. These findings are consistent with previous report on carriage of HLGR genes on transposons in enterococcus species (18, 19). This finding suggests that aminoglycoside resistance genes are possibly disseminated through the population of enterococci species by Tn5281.

In conclusion, the results of this study revealed high prevalence of HLGR genes among enterococcal isolates in the study region. The carriage of resistance gene on mobile element Tn5281 reminded the possibility of dissemination of HLGR among different pathogenic bacteria.

## References

[1] Karlowsky JA, Jones ME, Draghi DC, Thorns Berry C, Sahm DF, Volturo GA (2004). Prevalence and antimicrobial susceptibilities of bacteria isolated from blood cultures of hospitalized patients in the United States in 2002. Ann Clin Microbiol Antimicrob.

[2] Maki DG, Agger WA (1988). Enterococcal bacteremia: clinical features, the risk of endocarditis, and management. Medicine (Baltimore).

[3] Murray BE (1990). The life and times of the Enterococcus. Clin Microbiol Rev..

[4] Horodniceanu T, Bougueleret L, El-Solh N, Bieth G, Delbos F (2007). High level plasmid-borne resistance to gentamicin in *Streptococcus faecalis* subsp. Zymogenes. Antimicrob Agents Chemother.

[5] Mayer KH, Opal SM, Medeiros AA, Man dell G. L, Bennett J. E, Dolin R (2000). Mechanisms of antibiotic resistance. Principles and Practice of Infectious Diseases.

[6] Fisher K, Phillips C (2009). The ecology, epidemiology and virulence of Enterococcus. Microbiology.

[7] Sheppard BD, Gilmore MS (2002). Antibiotic-resistant enterococci: the mechanisms and dynamics of drug introduction and resistance. Microbes Infect.

[8] Thal LA, Chow JW, Patterson J (1993). Molecular characterization of highly gentamicin-resistant *enterococcus faecalis* isolates lacking high-level streptomycin resistance. Antimicrob Agents Chemother.

[9] Huycke MM, Sahm DF, Gilmore MS (1998). Multiple drug resistant enterococci: the nature of the problem and an agenda for the future. Emerg Infect Dis.

[10] Woodford N, Morrison D, Cookson B, George RC (1993). Comparison of high-level gentamicin resistant *Enterococcus faecium* isolates from different continents. Antimicrob Agents Chemother.

[11] Swenson JM, Ferraro MJ, Sahm DF, Clark NC, Culver DH, Tenover FC (1995). Multilaboratory evaluation of screening methods for detection of high-level aminoglycoside resistance in enterococci. J Clin Microbiol.

[12] Blanch AR (1999). Identification of Enterococcus spp. with a biochemical key. Appl Environ Microbiol.

[13] Clinical and laboratory standards institute (CLSI) (2009). Methods for dilution antimicrobial susceptibility tests for bacteria that grow. aerobically: Approved Standard __8th Edition. CLSI document M07-A8 (ISBN 1-56238-689-1).

[14] Regnault B (1997). Universal ribotyping methods using a chemically labelled oligonucleotide probe mixture. Res Microbiol.

[15] van de Klundert JAM, Vliegenthart JS, Persing D. H, Smith T. F, Tenover F.C, White T. J (1993). PCR detection of genes coding for amino glycoside-modifying enzymes. Diagnostic Molecular Microbiology: Principles and Applications.

[16] Woodford N, Mcnamara E, Smyth E, George RC (1992). High-level resistance to gentamicin in *enterococcus faecium*. J Antimicrob Chemother.

[17] Expand long template PCR system (2005). Deoxynucleoside-triphosphate:DNA deoxy nucleotidyl transferase.

[18] Zarrilli R, Tripodi MF, Fortunato R, Bagattini M (2005). Molecular epidemiology of high-level aminoglycoside-resistant enterococci isolated from patients in a university hospital in southern Italy. J Antimicrob Chemother.

[19] Arvanitidou M, Katsouyannopoulos V, Tsakris A (2001). Antibiotic resistance patterns of enterococci isolated from coastal bathing waters. J Med Microbiol.

[20] Simjee S, Gill MJ (1997). Gene transfer, gentamicin resistance and enterococci. J Hosp Infect.

[21] Mrenda G, Lee L, Kelly C, Solorzano F, Leaños B, Muñoz O (2001). Antimicrobial resistance from enterococci in a pediatric hospital. Plasmids in *Enterococcus faecalis* isolates with high level gentamicin and streptomycin resistance. Arch Med Res..

[22] Billstrom H, Lund B, Sullivan A, Nord CE (2008). Virulence and antimicrobial resistance in clinical *Enterococcus faecium*. Int J Antimicrob Agents.

[23] Patterson JE, Masecar BL, Kauffman CA, Schaberg DR, Hierholzer WJ, Zervos MJ (1988). Gentamicin resistance plasmids of enterococci from diverse geographic areas are heterogeneous. J Infect Dis.

[24] Dadfarma N, Oskouei M, Imani Fouladi AA, Farokh P (2010). Study of *aac(6′)ie-aph(2″6)ia* gene in clinical strain of enterococci and identification of high-level gentamicin resistante enterococci. Scientific Journal of Hamadan University of Medical Sciences and Health Services.

[25] Feizabadi MM, Shokrzadeh L, Sayady S, Asadi S (2008). Transposon Tn5281 is the main distributor of the aminoglycoside modifying enzyme gene among isolates of Enterococcus faecalis in Tehran hospitals. Can J Microbiol.

[26] Vigani AG, Macedo de Oliveira A, Bratfich OJ, Stucchi RSB, Moretti ML (2008). Clinical, epidemiological, and microbiological characteristics of bacteremia caused by high-level gentamicin-resistant *Enterococcus faecalis*. Braz J Med Biol Res.

[27] Vakulenko SB, Donabedian SM, Voskresenskiy AM, Voskresenskiy AM, Zervos MJ, Lerner SA (2003). Multiplex PCR for detection of aminoglycoside resistance genes in enterococci. Antimicrob Agents Chemother.

[28] Sahm DF, Gilmore MS (1994). Transferability and genetic relatedness of high-level gentamicin resistance among enterococci. Antimicrob Agents Chemother.

[29] Keddy KH, Klugman KP, Liebowitz LD (1996). Incidence of high-level gentamicin resistance in enterococci at Johannesburg Hospital. S Afr Med J.

[30] Papaparaskevas J, Vatopoulos A, Tassios PT, Avlami A, Legakis NJ, Kalapothaki V (2000). Diversity among high-level aminoglycoside-resistant enterococci. J Antimicrob Chemother.

